# Silencing of Long Non-Coding RNA LINC00607 Prevents Tumor Proliferation of Osteosarcoma by Acting as a Sponge of miR-607 to Downregulate E2F6

**DOI:** 10.3389/fonc.2020.584452

**Published:** 2021-01-28

**Authors:** Yuehuan Zheng, Zhe Chen, Zezhu Zhou, Xiangyang Xu, Huilin Yang

**Affiliations:** ^1^Department of Orthopedics, The First Affiliated Hospital of Soochow University, Suzhou, China; ^2^Department of Orthopedics, Ruijin Hospital, Shanghai Jiaotong University School of Medicine, Shanghai, China; ^3^Department of Orthopedics, Ruijin Hospital North, Shanghai Jiaotong University School of Medicine, Shanghai, China; ^4^Department of Orthopedics, Shanghai Tenth People’s Hospital, Tongji University, Shanghai, China

**Keywords:** lncRNA, *LINC00607*, osteosarcoma, miR-607, E2f6

## Abstract

Osteosarcoma (OS), a type of malignant bone tumor, is commonly found in children and adolescents. Although previous studies have identified that long non-coding RNAs (lncRNAs) regulate OS, it is unclear whether lncRNAs impact the progression of OS. Here, we identified *LINC00607*, a lncRNA that facilitates OS proliferation, migration, and invasion. Based on the RNA-sequencing results, *LINC00607* expression was significantly upregulated in pulmonary metastasis within OS. Functional experiments revealed that *LINC00607* promoted migration and invasion of endothelial cells to exacerbate epithelial-mesenchymal transition (EMT). Furthermore, the results of RNA pull-down assay and invasion assay suggested that the binding between *LINC00607* and miR-607 promoted OS invasion. Bioinformatic analysis and rescue experiments demonstrated that *E2F6*, a transcriptional factor, functioned downstream of *LINC00607*/miR-607. Finally, we found that *LINC00607* promoted OS progression *in vivo*. This work revealed that *LINC00607* worked as an miR-607 sponge to upregulate *E2F6* expression, which promoted tumor proliferation in OS. These results identified a novel therapeutic target for treating OS.

## Introduction

Osteosarcoma (OS) is the most common type of primary bone cancer, and it typically occurs in children and adolescents with a worldwide rate of incidence of 3.1 per million (1–4). It is mainly associated with height, birth weight, and germline genetic variants (5–9). The overall 5-year survival rate in patients with the non-metastatic disease and with the metastatic disease is 60–70 and 25%, respectively (10–12). Although less common than soft-tissue sarcoma, OS is challenging for both patients and their caregivers, and there have been very few advancements in treatment in the past few decades (13, 14). Thus, there is an urgent need to identify the molecules associated with metastasis as well as the corresponding mechanisms to develop therapeutic strategies.

Long non-coding RNAs (lncRNAs), a novel type of noncoding RNAs, contain >200 nucleotides and participate in several physiological processes, such as tumor initiation and progression through epigenetic, transcriptional, and post-transcriptional pathways (15–17). LncRNAs play multiple roles in various types of malignant tumors, such as lung adenocarcinoma (LUAD), hepatocellular carcinoma (HCC), and OS (18–22). Long intergenic non-protein coding RNA 00607 (*LINC00607*) is a novel lncRNA, which is significantly downregulated in LUAD (23). However, there are no published reports on the relationship between *LINC00607* and OS.

The epithelial-mesenchymal transition (EMT) plays a vital role in cancer progression and metastasis (24). Recent studies have shown that lncRNA (25) and miRNA (26) can regulate EMT post-transcriptionally. For OS, approximately 20% of patients are diagnosed with severe metastatic disease exhibit pulmonary metastases (60–70%) (27, 28). Thus, understanding the mechanisms of OS metastasis would facilitate the development of OS therapy.

Here, we found elevated levels of *LINC00607* in OS, which promoted OS progression *via* EMT. Further, we identified that *LINC00607* acted as an miR-607 sponge to modulate *E2F6* expression and directly regulated the *in vivo* tumor growth of OS.

## Method

### Cell Culture and Transfection

The human bone marrow mesenchymal stem cells (hBMSCs), human osteoblasts (hFOB1.19), and human OS cell lines (U2OS, Saos-2, MG63, and HOS) were purchased from the cell bank of the Chinese Academy of Sciences. These cells were cultured in the FBS-supplemented DMEM medium containing 1% penicillin-streptomycin (Hyclone, USA). We constructed overexpressed and silenced plasmids for LINC00607 and E2F6. We procured miR-607 mimics and inhibitors from GenePharma Co. Ltd. (Shanghai, China), which were transfected using Lipo3000 (Invitrogen).

### RNA Isolation and Quantitative Real-Time PCR (qRT-PCR)

The TRIzol reagent was used for total RNA extraction. Next, we used SuperScript III reverse transcriptase with random primer for mRNA to synthesize cDNA. RT-PCR was performed using SYBR Green (TAKARA, Japan). The ΔΔCt method was used for data analysis. Additionally, GAPDH was used for normalizing the data.

### Fluorescence *In Situ* Hybridization

We synthesized the probe for *LINC00607* using the Digoxigenin labeling mix (Roche, Germany), followed by fluorescence *in situ* hybridization. Briefly, after fixing in 4% paraformaldehyde and washing with 1× PBS containing 0.5% Triton X-100, the cells were kept in overnight incubation with the diluted probe at 37°C. Next, the samples were washed with the following solutions:2X SSC thrice for 5 min each, 0.2x SSC thrice for 10 min each, PBS-T (0.1% Tween in PBS) thrice for 5 min each. Then, the cells were kept in the 2% Blocking Reagent (Roche, Germany) for 1 h, followed by incubation in anti-Digoxigenin-POD Fab Fragments (Roche, Germany, 1:1,000). After washing with PBS, the samples were stained in a Cy3-containing staining buffer (1:50, PerkinElmer, USA) for 20 min.

#### Flow Cytometry Analysis

The cells were labeled with annexin V-FITC and PI for apoptotic analysis. Briefly, the cells were harvested at 48 h post-transfection with either control, LINC00607-overexpressing, or LINC00607-knockdown plasmids. Next, they were washed with cold PBS (1×), and resuspended in the binding buffer, and stained with annexin V-FITC and PI solution (BD Pharmingen, USA) at room temperature for 15 min in the dark. Post-incubation, we added the binding buffer (500 µl) and analyzed the cells using flow cytometry (BD Biosciences).

### Cell Proliferation, Migration, and Invasion Assay

Cellular proliferation was analyzed using the CCK-8 assay (DOJINDO, Japan). Post-transfection, the cells were plated in a 96-well plate and cultured for 12, 24, 48, 72, and 96 h, followed by measurement of absorbance at 450 nm using a microplate reader. We performed a scratch wound healing assay to evaluate the migration of the OS cells. Briefly, the cells (1 × 10^6^ per well) were seeded in a six-well plate, followed by transfection with the corresponding plasmids. Each well was scratched using a 200 µl pipette tip and incubated for 24 h. ImageJ was used to capture and analyze the wounded areas. We tested cell invasion by the Transwell assay, which was performed using a transwell chamber with 8 μm pores, which were coated with Matrigel (Sigma-Aldrich, USA). Cells were cultured in serum-free medium and 10% FBS-containing medium in the upper chamber and the lower chamber, respectively. After incubation for 48 h, the cells in the upper chamber were removed and the lower surface cells were stained with crystal violet. Then, we estimated the cell count based on the captured microscopic images.

### Cell Colony Formation Assay

For cell colony formation assay, cells were seeded in a six-well plate at 4,000 cells/cm^2^ and were cultured in 10% FBS-containing RPMI-1640 medium, followed by transfection with the corresponding plasmids. After 2 weeks, the cell colonies were stained with crystal violet and counted.

### Western Blotting

The cells were seeded in a six-well plate and lysed in lysis buffer (50 mM Tris-HCl pH 7.4, 150 mM NaCl, 0.1% SDS, 1% NP-40, 1 mM PMSF, and protease inhibitor cocktail). We collected protein fractions after centrifugation at 12,000 g at 4°C for 15 min. The samples were electrophoresed on a 10% SDS-PAGE and transferred to polyvinylidene difluoride (PVDF) membranes. The non-specific sites on the membrane were blocked using 5% BSA for 1 h at room temperature, followed by incubation with specific antibodies overnight at 4°C. An HRP-labeled secondary antibody was added and visualized by the enhanced chemiluminescence detection system (Millipore, Billerica, MA, USA). The following primary antibodies were used in this study: Bax (1:1,000, Abcam, #ab32503), E-cadherin (1:1,000, Abcam, #ab76055), Fibronectin (1:1,000, Abcam, #ab2413), TWIST (1:1,000, Abcam, #ab175430), Vimentin (1:1,000, Abcam, #ab92547), VEGF (1:1,000, Abcam, #ab72807), E2F6 (1:1,000, Abcam, #ab155978), and GAPDH (1:1,000, Abcam, #ab8245).

### RNA Pull-Down Assay

MS2 binding sites (MS2bs) are RNAs (approximately 19 bp long) that can bind MS2-coated proteins with their specific stem-loop structure. Briefly, U2OS cells were co-transfected with pcDNA3-Flag-MS2bp and either LINC00607-MS2bs or NC-MS2bs, treated with the IP lysis buffer, followed by harvesting the cell lysates after 48 h. Anti-FLAG^®^ M2 Magnetic Beads was used to immunoprecipitate Flag-MS2bp fusion protein. The co-precipitated RNAs related to Flag-MS2bp were extracted using the Trizol reagent and identified by qRT-PCR. Next, the probe was incubated with Dynabeads M-280 Streptavidin (Invitrogen, USA) for 10 min at room temperature to obtain the probe-coated beads, which were then incubated with U2OS cell lysates. After washing the incubated beads, the TRIzol reagent was added to the sample, and RNA was extracted. Next, *LINC00607* expression was detected through RT-PCR.

### Dual-Luciferase Assay

The wild-type or mutated binding sites of miR-607 in *LINC00607* or *E2F6* were inserted into the downstream of the firefly luciferase gene in a psiCHECK-2 vector (Promega, USA). Next, the corresponding plasmids and microRNA mimics were transfected into the 293T cells. Post-incubation for 48 h, the luciferase activity was measured using the dual-luciferase reporter assay system (Promega) on a luminometer. Luciferase activity was normalized by Renilla luciferase activity.

### Tumor Formation

The experimental protocol was approved by the experimental animal ethics committee of the First Affiliated Hospital of Soochow University and was conducted following the institutional animal care guidelines. We procured C57BL/6 J nude mice (6-weeks-old; female) from Shanghai SLAC Laboratory Animal Co. Ltd. and raised them in an SPF room with a 12 h light/dark cycle. U2OS cells were injected into the flanks of female WT nude mice. Thirty days post-injection, the mice were anesthetized, and the tumors were removed for weight measurement.

### Statistical Analysis

All results were calculated based on at least three independent experiments, and the data were presented as mean ± S.D. All statistical analyses were performed with SPSS v13.0. Statistical differences between two groups were determined using the Student’s *t*-test, and among groups were analyzed by one-way analysis of variance (ANOVA) with a *post-hoc* test. Differences were considered significant when *p*-value <0.05.

## Results

### LINC00607 Was Upregulated in OS

**Figure 1:**
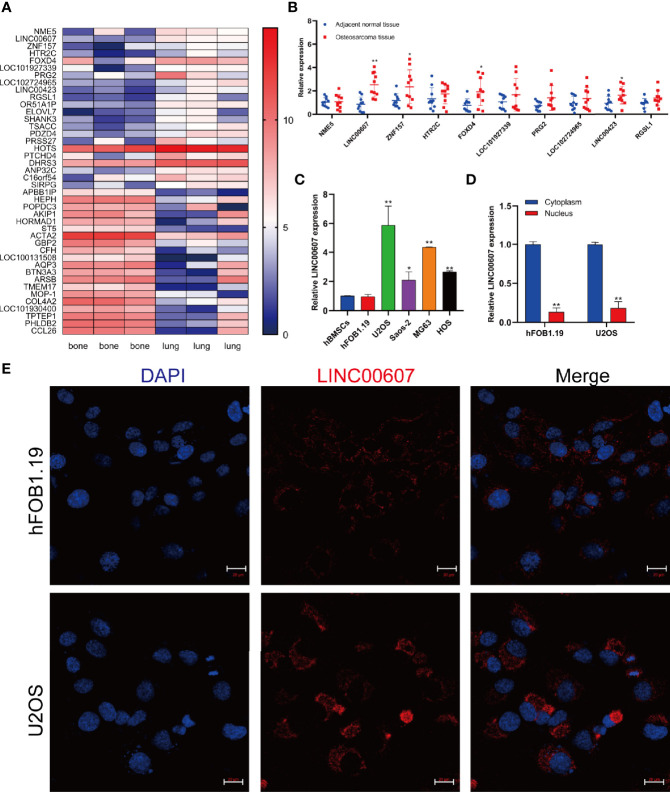
*LINC00607* was upregulated in OS. **(A)** Heatmap of the upregulated genes in lung metastases, as reported in GEO (GSE85537). **(B)** Relative expression of the top 10 genes in adjacent normal tissues and OS tissues. **(C)** Relative expression of *LINC00607* in hBMSCs, hFOB1.19, as well as U2OS, Saos-2, MG63, and HOS cell lines by RT-PCR. **(D)** Quantification of *LINC00607* expression in the cytoplasmic fractions and nuclear fractions of Hfob1.19 and U2OS cells by RT-PCR. **(E)** Fluorescence *in situ* hybridization (FISH) of *LINC00607* in hFOB1.19 and U2OS cells. Statistical analysis was conducted using Student’s *t*-test. Values are represented as mean ± SD compared with the control group. *p < 0.05, **p < 0.01.

It is vital to investigate metastasis-related molecules to identify potential targets to optimize OS and its treatment (29). We integrated the recently published RNA-sequencing database of patients with OS (GEO Dataset: GSE85537) and found that 42 genes were upregulated both in the bone and in the lungs (**Figure 1A**). We focused on the top 10 upregulated genes and found significantly upregulated expression of *LINC00607*, *ZNF157*, *FOXD4*, and *LINC00423* in OS tissue compared with adjacent normal tissue (**Figure 1B**). Since *LINC00607* showed the highest expression, we evaluated its expression in several human OS cell lines. The RT-PCR results showed that *LINC00607* was highly expressed in the U2OS and MG63 cell lines (**Figure 1C**) and mainly in the cytoplasm (**Figure 1D**). The fluorescence *in situ* hybridization (FISH) of *LINC00607* in hFOB1.19 and U2OS showed a similar pattern (**Figure 1E**). These data demonstrated that *LINC00607* was upregulated in OS and was mainly located in the cytoplasm.

### Overexpression of LINC00607 Promoted Cell Proliferation, Migration, and Invasion

**Figure 2:**
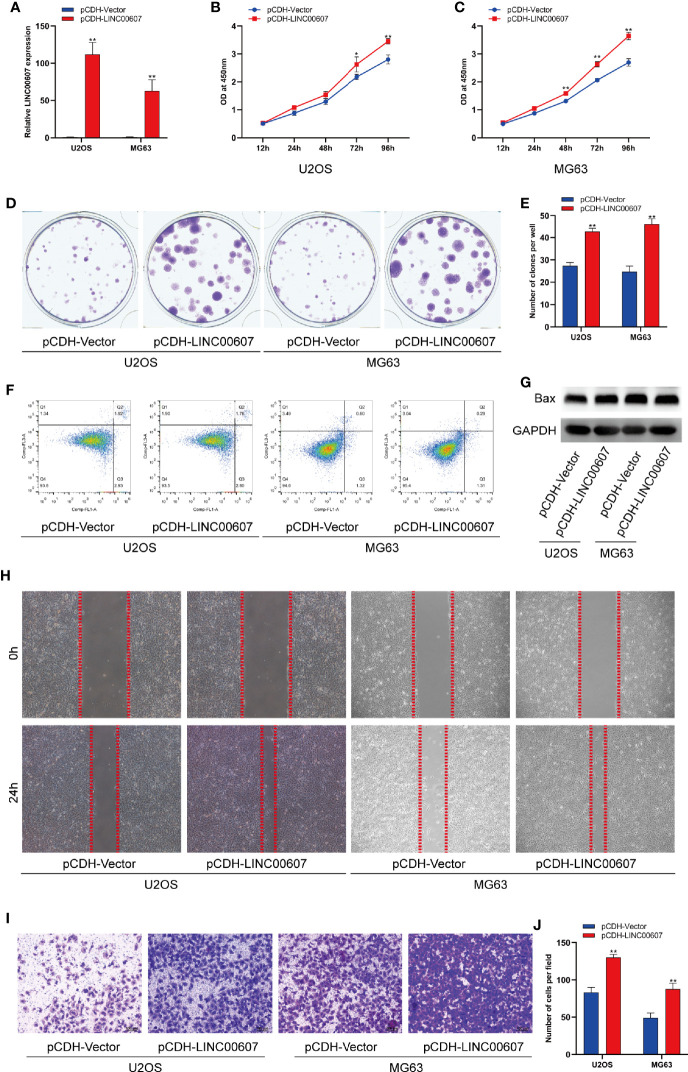
Overexpression of *LINC00607* promoted cell proliferation, migration, and invasion. **(A)** Relative expression of *LINC00607* in U2OS and MG63 cells co-transfected with pCDH-vector and pCDH-LINC00607 by RT-PCR. **(B)** Cellular proliferation was evaluated by CCK-8 assay in U2OS cells transfected with either control or overexpressing plasmids. **(C)** Cellular proliferation was evaluated by CCK-8 assay in MG63 cells transfected with either control or overexpressing plasmids. **(D)** Colony formation was visualized through crystal violet staining in U2OS and MG63 cells transfected with either control or overexpressing plasmids. **(E)** Quantification of clone number/well in **(D, F)** Apoptotic analysis of U2OS and MG63 cells transfected with either control or overexpressing plasmids by FACS. **(G)** Detection of Bax and GAPDH in U2OS and MG63 cells transfected with either control or overexpressing plasmids through Western Blot. **(H)** Assessment of the migration ability of U2OS and MG63 OS cells transfected with either control or overexpressing plasmids by scratch wound healing assay. **(I)** Assessment of the invasive ability of U2OS and MG63 OS cells transfected with either control or overexpressing plasmids by Transwell assay. **(J)** Quantification of the number of cells/field in **(I)** Statistical analysis was conducted using Student’s *t*-test. Values are represented as mean ± SD compared with the control group. *p < 0.05, **p < 0.01.

Next, we explored the potential role of *LINC00607* in the proliferation, migration, and invasion of OS cell lines. We successfully overexpressed *LINC00607* in U2OS and MG63 cell lines (**Figure 2A**) and found that LINC00607-overexpressing plasmids-transfected cell lines were more proliferative than the control group, based on the results of the CCK-8 assay (**Figures 2B, C**). The cell colony formation assay showed similar results (**Figures 2D, E**). Next, we conducted flow cytometry to examine U2OS or MG63 cells transfected with LINC00607-overexpressing plasmids to assess whether these effects were associated with cellular apoptosis and found that the proportion of apoptotic cells were comparable with the control groups (**Figure 2F**). Additionally, we observed that the protein expression of Bax was unaffected in LINC00607-overexpressing cells (**Figure 2G**). These results indicated that overexpression of *LINC00607* had a minor impact on cellular apoptosis. Scratch wound healing assay indicated that U2OS or MG63 cells transfected with LINC00607-overexpressing plasmids had a remarkable migration ability (**Figure 2H**). The invasion assay showed that LINC00607-overexpressing cells possessed a much stronger ability to invade through Matrigel than the control cells (**Figures 2I–J**). Thus, these results demonstrated that *LINC00607* promoted OS cell proliferation, migration, and invasion *in vitro*.

### Knockdown of LINC00607 Inhibited Cell Proliferation, Migration, and Invasion

**Figure 3:**
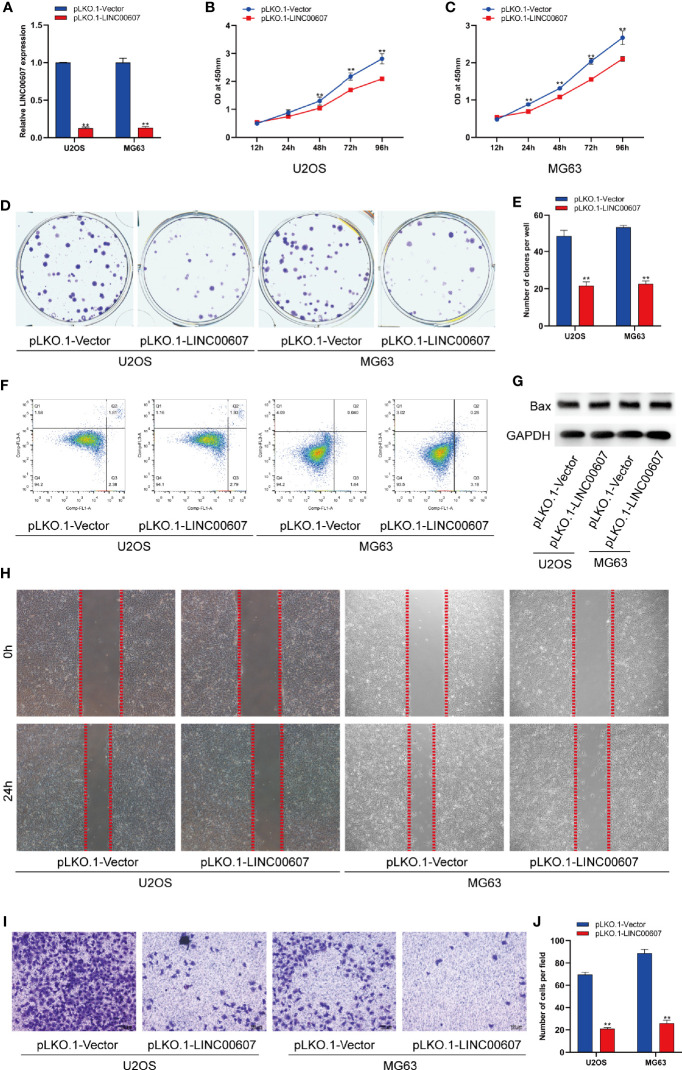
Knockdown of *LINC00607* inhibited cell proliferation, migration, and invasion. **(A)** Relative expression of *LINC00607* in U2OS and MG63 cells co-transfected with pLKO.1-vector and pLKO.1-LINC00607 by RT-PCR. **(B)** Cellular proliferation was evaluated by CCK-8 assay in U2OS cells transfected with either control or knockdown plasmids. **(C)** Cellular proliferation was evaluated by CCK-8 assay using in MG63 cells transfected with either control or knockdown plasmids. **(D)** Colony formation was visualized through crystal violet staining in U2OS and MG63 cells transfected with either control or knockdown plasmids. **(E)** Quantification of clone number/well in **(D, F)** Apoptotic analysis of U2OS and MG63 cells transfected with either control or knockdown plasmids by FACS. **(G)** Detection of Bax and GAPDH in U2OS and MG63 cells transfected with either control or knockdown plasmids through Western Blot. **(H)** Assessment of the migration ability of U2OS and MG63 OS cells transfected with either control or knockdown plasmids by scratch wound healing assay. **(I)** Assessment of the invasion ability of U2OS and MG63 OS cells transfected with either control or knockdown plasmids by Transwell and crystal violet assay. **(J)** Quantification of the number of cells/field in **(I)** Statistical analysis was conducted using Student’s *t*-test. Values are expressed as mean ± SD compared with the control group. **p < 0.01.

The results of the loss-of-function study revealed that U2OS and MG63 cells transfected with *LINC00607*-silenced plasmid (**Figure 3A**) exhibited less proliferation than the control group by CCK-8 assay (**Figures 3B, C**). Unlike overexpression, knockdown of *LINC00607* inhibited the proliferation of U2OS and MG63 cells (**Figures 3D, E**). Additionally, cellular apoptosis was also unaffected in the *LINC00607*-knockdown cell lines (**Figures 3F, G**). We observed reduced migration ability post-*LINC00607* knockdown based on the results of the scratch wound healing assay (**Figure 3H**). Similarly, the invasive ability was also weakened in U2OS and MG63 cells after *LINC00607* knockdown (**Figures 3I, J**). Thus, these data demonstrated that knockdown of *LINC00607* in U2OS and MG63 cell lines decreased OS cell proliferation, migration, and invasion.

### Overexpression of LINC00607 Influenced EMT and Promoted the Migration and Invasion of Endothelial Cells

**Figure 4:**
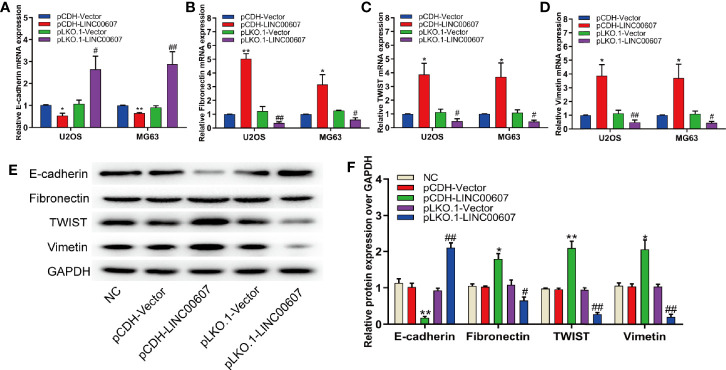
Overexpression of *LINC00607* influenced EMT. **(A)** Quantification of *E-cadherin* mRNA expression in U2OS and MG63 cells post-overexpression/knockdown of *LINC00607* by RT-PCR. **(B)** Quantification of *Fibronectin* mRNA expression in U2OS and MG63 cells post-overexpression/knockdown of *LINC00607* by RT-PCR. **(C)** Quantification of *TWIST* mRNA expression in U2OS and MG63 cells post-overexpression/knockdown of *LINC00607* by RT-PCR. **(D)** Quantification of *Vimentin* mRNA expression in U2OS and MG63 cells post-overexpression/knockdown of *LINC00607* by RT-PCR. **(E)** Detection of E-cadherin, Fibronectin, TWIST, and Vimentin in U2OS cells post-overexpression/knockdown of *LINC00607* through Western Blot. **(F)** Quantitative analysis of E-cadherin, Fibronectin, TWIST, and Vimentin in U2OS cells post-overexpression/knockdown of *LINC00607* through Western Blot. Statistical analysis was conducted using Student’s *t*-test. Values are expressed as mean ± SD compared with the control group. (*: pCDH-LINC00607 *vs.* pCDH-vector) *p < 0.05, **p < 0.01; (#: pLKO.1-LINC00607 *vs.* pLKO.1-vector), ^#^*p* < 0.05, ^##^*p* < 0.01.

**Figure 5:**
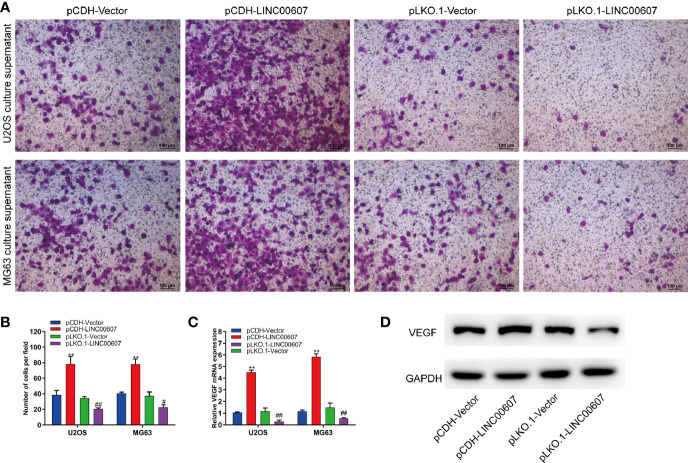
Overexpression of *LINC00607* promoted the migration and invasion of endothelial cells. **(A)** Evaluation of the invasive ability of endothelial cells cultured with the supernatants of U2OS or MG63 cells with *LINC00607* overexpression/knockdown by Transwell and crystal violet assay. **(B)** Quantification of the number of cells/field in **(A, C)** Relative mRNA expression of *VEGF* in endothelial cells cultured with the supernatants of U2OS or MG63 cells with *LINC00607* overexpression/knockdown by RT-PCR. **(D)** Detection of VEGF and GAPDH in endothelial cells cultured with the supernatants of U2OS cells with *LINC00607* overexpression/knockdown by Western blotting. Statistical analysis was conducted using Student’s *t*-test. Values are expressed as mean ± SD compared with the control group. (*: pCDH-LINC00607 *vs.* pCDH-vector) **p < 0.01; (#: pLKO.1-LINC00607 *vs.* pLKO.1-vector) ^#^*p* < 0.05, ^##^*p* < 0.01.

Since approximately 25% of OS patients have pulmonary metastasis when diagnosed (27), and since EMT is important for the metastatic dissemination of tumor cells, we investigated whether any change in the expression of *LINC00607* affected EMT. The results of RT-PCR and western blot revealed that the overexpression of *LINC00607* inhibited the expression of E-cadherin (**Figures 4A, E**) and promoted the expression of fibronectin, TWIST, and vimentin (**Figures 4B–F**). As expected, *LINC00607* knockdown showed opposite results. Since tumor cells can secrete molecules that facilitate EMT, we tested the medium supernatants of U2OS and MG63 cells. The results of the invasion assay demonstrated that *LINC00607*-overexpressed supernatants of both U2OS and MG63 cells considerably accelerated the invasion of endothelial cells, while the *LINC00607*-knockdown supernatants inhibited it (**Figures 5A, B**). Additionally, the *LINC00607*-overexpressed supernatants promoted the expression of VEGF, a vital factor for vasculogenesis and angiogenesis, and *LINC00607*-knockdown supernatants inhibited VEGF expression (**Figures 5C, D**). Overall, these data demonstrated that *LINC00607* induced EMT *in vitro*, and promoted the migration and invasion of endothelial cells.

### LINC00607 Regulated OS Invasion Through Sponging miR-607

**Figure 6:**
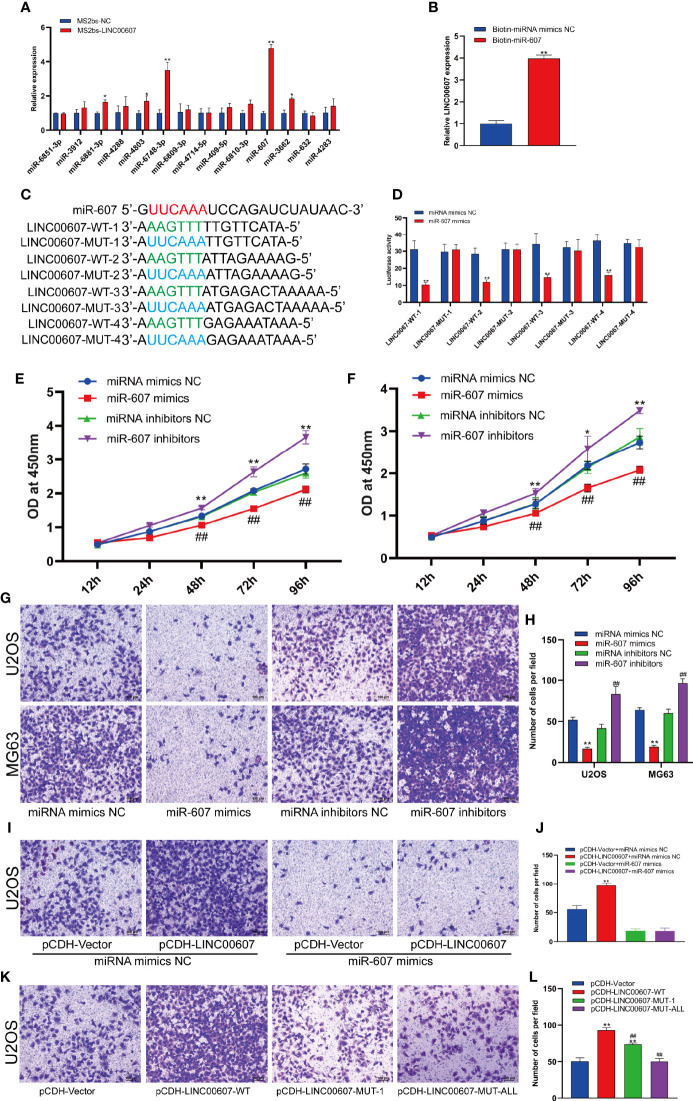
*LINC00607* regulated OS invasion through sponging miR-607. **(A)** Relative expression of miRNA candidates in MS2bs-NC or MS2bs-LINC00607 RNA pull-down sample. **(B)** Relative expression of *LINC00607* in biotin-miRNA mimics NC and biotin-miR-607 pull-down samples. **(C)** The sequences of miR-607, wild-type, and mutant binding sites in *LINC00607*. **(D)** The effect of miRNA mimics NC or miR-607 mimics on wild-type or mutant binding sites in *LINC00607* by luciferase assay. **(E)** The dynamic expression of *LINC00607* in U2OS cells with overexpressed/knockdown miR-607 at 12, 24, 48, 72, and 96 h by RT-PCR. **(F)** The expression of *LINC00607* in MG63 cells with overexpressed/knockdown miR-607 at 12, 24, 48, 72, and 96 h by RT-PCR. **(G)** Assessment of the invasive ability of U2OS and MG63 OS cells transfected with mimics NC, miR-607, inhibitors NC, or miR-607 inhibitors in U2OS cells by Transwell and crystal violet assay. **(H)** Quantification of the number of cells/field in **(G, I)** Assessment of the invasive ability of U2OS cells co-transfected with mimics NC with control or *LINC00607*-overexpressing plasmids and miR-607 mimics with control or *LINC00607*-overexpressing plasmids by Transwell and crystal violet assay. **(J)** Quantification of the number of cells/field in **(I, K)** Assessment of the invasive ability of U2OS cells transfected with control plasmids, wild-type *LINC00607*-overexpressing plasmids, LINC00607-overexpressing plasmids with one mutated binding site or *LINC00607*-overexpressing plasmids with all binding sites mutated by Transwell and crystal violet assay. **(L)** Quantification of the number of cells/field in **(K)** Statistical analysis was conducted using Student’s *t*-test. Values are expressed as mean ± SD compared with the control group. *p < 0.05, **p < 0.01, ^##^*p* < 0.01.

LncRNAs are known to regulate miRNAs by acting as competing endogenous RNAs (30, 31). It is unclear whether *LINC00607* functions using the same approach. Based on the cytoplasmic location of *LINC00607*, we predicted that there were 14 miRNA candidates with the potential to interact with *LINC00607* through miRDB. Thus, we performed an RNA pull-down assay to confirm these predictions and found that the level of miR-607 in the MS2bs-LINC00607 pull-down sample was remarkably higher than that in the control sample (**Figure 6A**). The results of these reverse validation experiment showed that *LINC00607* was detected in the biotin-miR-607 pull-down sample and was significantly higher compared with the control sample (**Figure 6B**). Bioinformatics prediction showed that *LINC00607* possessed four binding sites (**Figure 6C**). Also, the luciferase activity was significantly reduced in groups co-transfected with miR-607 mimics and LINC00607-WT reporter plasmids than the control group, while was unaffected in the case of mutated miR-607 binding sites (**Figure 6D**). Overexpression of miR-607 in U2OS and MG63 cell lines resulted in decreased expression of *LINC00607*, while knockdown of miR-607 promoted its expression (**Figures 6E–F**). Thus, these results revealed that there was a relationship between *LINC00607* and miR-607, and miR-607 regulated *LINC00607* expression.

Next, we performed the Transwell assay to verify the role of miR-607 in OS invasion and found that miR-607 overexpression decreased the invasive ability of OS cell lines, and miR-607 knockdown dramatically increased it (**Figures 6G, H**). Combining these results, we hypothesized that *LINC00607* participated in OS invasion *via* miR-607. The results of the Transwell assay revealed that if one binding site was mutated in *LINC00607*, it reduced the invasive ability of U2OS cells, while, if all binding sites were mutated in *LINC00607*, then, it restored the cells nearly to a normal state (**Figures 6K, L**). Then, we performed a rescue experiment, and the data showed that there was a significant decrease in the invasion ability of U2OS cells co-transfected with LINC00607-overexpressing plasmids and miR-607 mimics compared with the cells co-transfected with LINC00607-overexpressing plasmids alone (**Figures 6I, J**). Thus, LINC00607 regulated OS invasion by targeting miR-607 directly.

### LINC00607/miR-607 Modulated the Expression of E2F6

**Figure 7:**
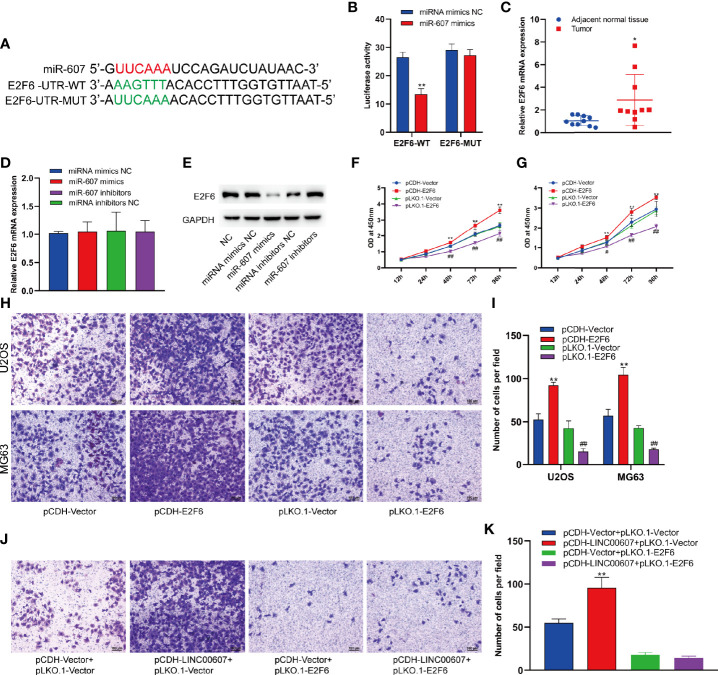
*LINC00607*/miR-607 modulated the expression of E2F6. **(A)** The sequence of wild-type miR-607and mutated miR-607 binding sites in *E2F6* UTR. **(B)** The effect of miRNA mimics NC or miR-607 mimics on the wild type or mutant binding sites in *E2F6* UTR by luciferase assay. **(C)** Relative expression of *E2F6* in the adjacent normal tissues and tumor by RT-PCR. **(D)** Relative expression of *E2F6* mRNA in U2OS with overexpressed/knockdown miR-607 by RT-PCR. **(E)** Detection of *E2F6* and *GAPDH* in U2OS cells with overexpressed/knockdown miR-607 through Western Blotting. **(F)** The dynamic expression of *LINC00607* in U2OS cells with overexpressed/knockdown *E2F6* at 12, 24, 48, 72, and 96 h by RT-PCR. **(G)** The dynamic expression of *LINC00607* in MG63 cells with overexpressed/knockdown *E2F6* at 12, 24, 48, 72, and 96 h by RT-PCR. **(H)** Assessment of the invasive ability of U2OS and MG63 cells transfected with control plasmids, E2F6-overexpressing/knockdown plasmids by Transwell and crystal violet assay. **(I)** Quantification of the number of cells/field in **(H, J)** Assessment of the invasive ability of U2OS cells co-transfected with either control or LINC00607-overexpressing plasmids with control or *E2F6*-silencing plasmids by Transwell and crystal violet assay. **(K)** Quantification of the number of cells/field in **(J)** Statistical analysis was conducted using Student’s *t*-test. Values are expressed as mean ± SD compared with the control group. *p < 0.05, **p < 0.01, ^#^*p* < 0.05, ^##^*p* < 0.01.

Since *LINC00607* is an endogenous sponge of miR-607, the target genes of miR-607 would play critical roles in OS invasion. Thus, we used TargetScan to predict the target genes and found a binding site of miR-607 in the 3’-UTR of E2F6, which played an important role in cell-cycle regulation (32). The results of luciferase reporter assay showed significant inhibition of luciferase activity by miR-607 mimics with wild-type UTR of E2F6, while the activity was unaffected for miR-607 mimics with mutant UTR of E2F6 (**Figures 7A, B**). The results of RT-PCR showed that the luciferase mRNA expression was unaffected by miR-607 (**Figure S1**). Next, we examined the *E2F6* expression in tumor tissues by RT-PCR to understand the role of *E2F6* in OS and found that its expression was relatively higher than the adjacent normal tissue sample (**Figure 7C**). The mRNA expression of *E2F6* was unaffected by overexpression or knockdown of miR-607 (**Figure 7D**), but the overexpression/knockdown of miR-607 resulted in a decreased/increased E2F6 protein expression (**Figure 7E**). RT-PCR detection showed that *LINC00607* did not affect E2F6 mRNA expression in U2OS cells (**Figure S2A**). However, *LINC00607* overexpression/knockdown promoted/inhibited E2F6 protein expression in U2OS cells (**Figure S2B**). The promotional effect of *LINC00607* on E2F6 protein expression was significantly reduced when the miR-607 expression was inhibited by miR-607 inhibitors (**Figure S2C**). Thus, we performed the RIP assay on Ago2, the core component of the RNA-induced knockdown complex, to confirm the role of miRNAs. Overexpression of *LINC00607* in U2OS cells led to increased enrichment of Ago2 on *LINC00607* but substantially decreased the enrichment on E2F6 transcripts (**Figure S2D**). However, *LINC00607* knockdown in U2OS cells had the opposite effect (**Figure S2E**). These results suggested that *LINC00607* could compete with E2F6 transcripts for the Ago2-based miRNA-induced repression complex. Overexpression/knockdown of *LINC00607* promoted/inhibited the expression of CDK6 (**Figure S5**). However, overexpression/knockdown of *LINC00607* inhibited/promoted the expression of p21 and BRCA1 in U2OS cells (**Figure S5**). Additionally, we found that *E2F6* overexpression/knockdown promoted/suppressed *LINC00607* expression in U2OS and MG63 cells (**Figure S4**). Both U2OS and MG63 cell lines showed that the overexpression/knockdown of E2F6 promoted/suppressed cellular proliferation (**Figures 7F, G**). These results indicated that *LINC00607* acted as a ceRNA to modulate *E2F6* expression. We performed the Transwell assay to confirm the role of *E2F6* and found that *E2F6* overexpression promoted the invasion ability of U2OS and MG63 cell lines, while *E2F6* knockdown impaired it (**Figures 7H, I**). Furthermore, *E2F6* knockdown counteracted the promotional effects of *LINC00607* overexpression on OS invasion (**Figures 7J, K**). Thus, these results demonstrated that *LINC00607*/miR-607 regulated OS invasion by modulating *E2F6* expression.

### LINC00607 Influenced the Tumor Growth of OS

**Figure 8:**
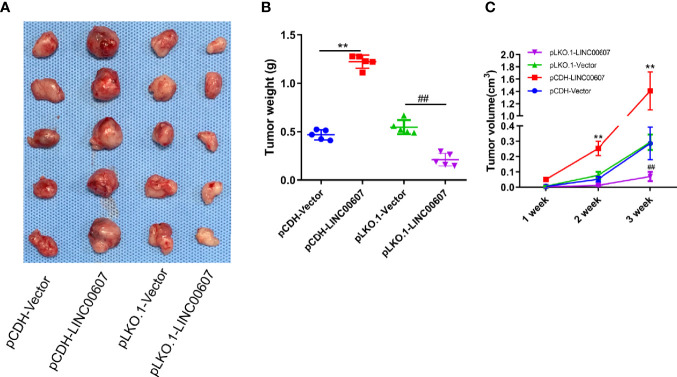
*LINC00607* influences the tumor growth of OS. **(A)** Images of the OS cell-derived xenograft tumors by subcutaneously injecting OS cell-overexpressing or silencing *RT* (n = 5). **(B)** Statistical analysis of the tumor weight in **(A, C)** Statistical analysis of tumor volume in **(A)** Statistical analysis was conducted using Student’s *t*-test. Values are expressed as mean ± SD compared with the control group. **p < 0.01, ^##^*p* < 0.01.

We injected pCDH-LINC00607 and pLKO-1-LINC00607 OS cells into the flanks of female WT nude mice to establish a subcutaneous tumor model to further investigate if *LINC00607* could affect tumor growth *in vivo*. We found that pCDH-LINC00607 cell-derived xenograft tumors grew faster than the control group, and pLKO-1-LINC00607 cell-derived xenograft tumors showed much slower growth (**Figure 8A**). There was a significant difference in weight and volume of the treated and control group mice (**Figures 8B, C**). This indicated that *LINC00607* affected *in vivo* OS tumor growth directly.

## Discussion

Here, we demonstrated that *LINC00607* facilitated OS proliferation, migration, and invasion by acting as an miR-607 sponge. Functional experiments indicated that *LINC00607* exacerbates EMT by promoting the migration and invasion of endothelial cells. Furthermore, we elucidated that the *LINC00607*/miR-607 axis modulated *E2F6*expression to influence the invasion of OS. Finally, *LINC00607* promoted OS tumor growth *in vivo*.

LncRNAs are known to play crucial roles in cancer cell regulation (33). As a novel lncRNA, the role of *LINC00607* is poorly understood. Until now, only one study has mentioned a downregulated expression of *LINC00607* in LUAD (23); however, we found that its expression was upregulated in OS (**Figures 1A, B**). LncRNAs have different mechanisms in different types of tumors, which implies that they can have different effects in different types of tumors. The same lncRNA might act as a tumor suppressor gene or a proto-oncogene in different tumors. *LINC00607* is a tumor suppressor gene in LUAD and a proto-oncogene in OS. Thus, lncRNAs play diverse roles; for example, *H19* can act as either an oncogene or a tumor suppressor in HCC. Matouk et al. reported that in the Hep3B HCC cell line, hypoxic stress promoted *H19* expression and that H19 knockdown inhibited tumorigenicity after the cells were subcutaneously injected into nude mice (34). On the contrary, Zhang *et al. *showed a tumor suppressor role of *H19* (35). *LINC00607*, a species-specific lncRNA, exists only in humans, which restricts the study of its functional mechanism. Here, we initially investigated its role using *in vitro* models. With the recent advancements in technology, multiple therapeutic strategies have been developed to target lncRNAs (36–38), which provides a possibility that *LINC00607* also acts as a potential therapeutic target for OS.

Increasing evidence has shown that lncRNAs are related to subcellular localization (29). FISH analysis revealed that *LINC00607* was located in the cytoplasm, which indicated that it might regulate miRNAs by acting as ceRNAs. Based on this hypothesis, we found that *LINC00607* interacted with miR-607 and possessed four miR-607 binding sites. On mutating the miR-607 binding sites in *LINC00607*, we observed the inhibition of OS cell invasion. These results confirmed that *LINC00607* acted as an miR-607 sponge during OS development and progression. Previous studies on miR-607 have shown that excessive miR-607 decreases the proliferation, migration, and invasion of HeLa and CaSki cells (39), which contradicted its effect on OS cells. This inconsistency could be attributed to different types of tumors, which suggests that there is a unique mechanism involved in the development of OS. Also, there may exist more possible regulatory mechanisms of *LINC00607* besides miRNA sponge in OS development, which need further exploration.

In the past decade, studies have revealed that EMT and its intermediate states promote tumor progression (40). In our results, we demonstrated that *LINC00607* exacerbated the EMT process, promoted the migration and invasion of endothelial cells. Meanwhile, we found that *LINC00607* promoted VEGF expression. However, the detailed interaction mechanisms between OS and endothelial cells were unclear. Recent studies have revealed the role of OS-derived exosomes in tumor progression (41, 42). Secretory miR-25-3p was found to be embedded in OS-derived exosomes and promoted capillary formation as well as the invasion of vascular endothelial cells (41). One recent study also demonstrated that OS-derived exosomes promoted the differentiation of osteoclasts and potentiated tube formation in endothelial cells (42). Thus, understanding the underlying regulatory mechanism of the effects of *LINC00607* on endothelial cells would help in understanding OS progression. With the widespread application of transcriptomics and proteomics (43, 44), the unknown interaction between OS-derived exosomes and endothelial cells needs further investigation.

Previous studies regarding cell-cycle transcription factors *E2F6* revealed its involvement in the development and regulation of several genes involved in chromatin remodeling (45). *E2F6* is found in all types of OS tumors and displays nuclear co-localization in a punctate pattern in differentiating and confluent cells (46). Apart from a few studies on its promoter region (45), the detailed biological function and regulation of *E2F6* in OS development remains obscure. Here, we verified that *E2F6* promoted the invasion of OS cells, and *LINC00607*/miR-607 modulated *E2F6* expression through 3’-UTR. These mechanisms are mainly dependent on the degree of complementarity between miRNA and the target gene mRNA sequence. If miRNA and target gene mRNA are completely complementary, then miRNA regulates the target gene through cleavage; if miRNA and target gene mRNA have low complementarity, then miRNA regulates the target gene by inhibiting translation, which may require several miRNA molecules. In animals, most miRNAs have low complementarity with the mRNA of the target genes; thus, they regulate target genes by inhibiting translation. In this study, we found that miR-607 and *E2F6* 3‘-UTR regions were not completely complementary to each other. Thus, miR-607 inhibited the transcription of *E2F6* mRNA and did not affect the expression of *E2F6* mRNA. These results explained the function and regulatory mechanism of the *LINC00607*/miR-607/E2F6 axis in OS. However, there are more than one functional downstream effectors of *LINC00607*/miR-607, and high-throughput transcriptomics may help discover a more comprehensive regulatory network.

Thus, our findings provide the *LINC00607*/miR-607/E2F6 axis as a novel lncRNA-related pathway to control tumor proliferation in OS, which provides novel potential therapeutic targets for OS.

## Data Availability Statement

The raw data supporting the conclusions of this article will be made available by the authors, without undue reservation.

## Ethics Statement

The animal study was reviewed and approved by the experimental animal ethics committee of the First Affiliated Hospital of Soochow University.

## Author Contributions

HY and XX: Put forward the concept and design the experiment. YZ and ZC: did the experiment and analyzed the data. ZZ: article writing. All authors contributed to the article and approved the submitted version.

## Conflict of Interest

The authors declare that the research was conducted in the absence of any commercial or financial relationships that could be construed as a potential conflict of interest.

## Supplementary Material

Supplementary Figure 1 The effect of miRNA mimics NC or miR-607 mimics on the expression of luciferase mRNA with wild-type or mutant binding sites.

Supplementary Figure 2 *LINC00607* regulated E2F6 expression *via* miR-607. **(A)** RT-PCR detected E2F6 mRNA expression in U2OS cells with *LINC00607*-overexpressing/knockdown plasmids. **(B)** Western blot detected the expression of E2F6 protein with *LINC00607*-overexpressing/knockdown in U2OS cells. **(C)** Western blot detected the expression of E2F6 protein in U2OS cells with *LINC00607*-overexpressing plasmids and miR-607 inhibitors. **(D)** RIP assay of the enrichment of Ago2 on *LINC00607* and E2F6 transcripts relative to IgG in U2OS cells transfected with either pCDH-Vector or pCDH-LINC00607. **(E)** RIP assay of the enrichment of Ago2 on *LINC00607* and E2F6 transcripts relative to IgG in U2OS cells transfected with either pLKO.1-Vector or pLKO.1-LINC00607. Statistical analysis was conducted using Student’s *t*-test. Values are expressed as mean ± SD compared with the control group. **p* < 0.05, ***p* < 0.01.

Supplementary Figure 3 Fluorescence *in situ* hybridization (FISH) for *LINC00607* using sense sequence in U2OS.

Supplementary Figure 4 *E2F6* regulates the expression of *LINC00607* in U2OS and MG63 cells.

Supplementary Figure 5 *LINC00607* regulates the expression of p21, BRCA1, and CDK6.
