# Supplementary figures and images for: Silencing of Long Non-Coding RNA LINC00607 Prevents Tumor Proliferation of Osteosarcoma by Acting as a Sponge of miR-607 to Downregulate E2F6
Source: Front Oncol. 2021 Jan 28;10:584452. doi: 10.3389/fonc.2020.584452 (PMC7877452; doi:10.3389/fonc.2020.584452)

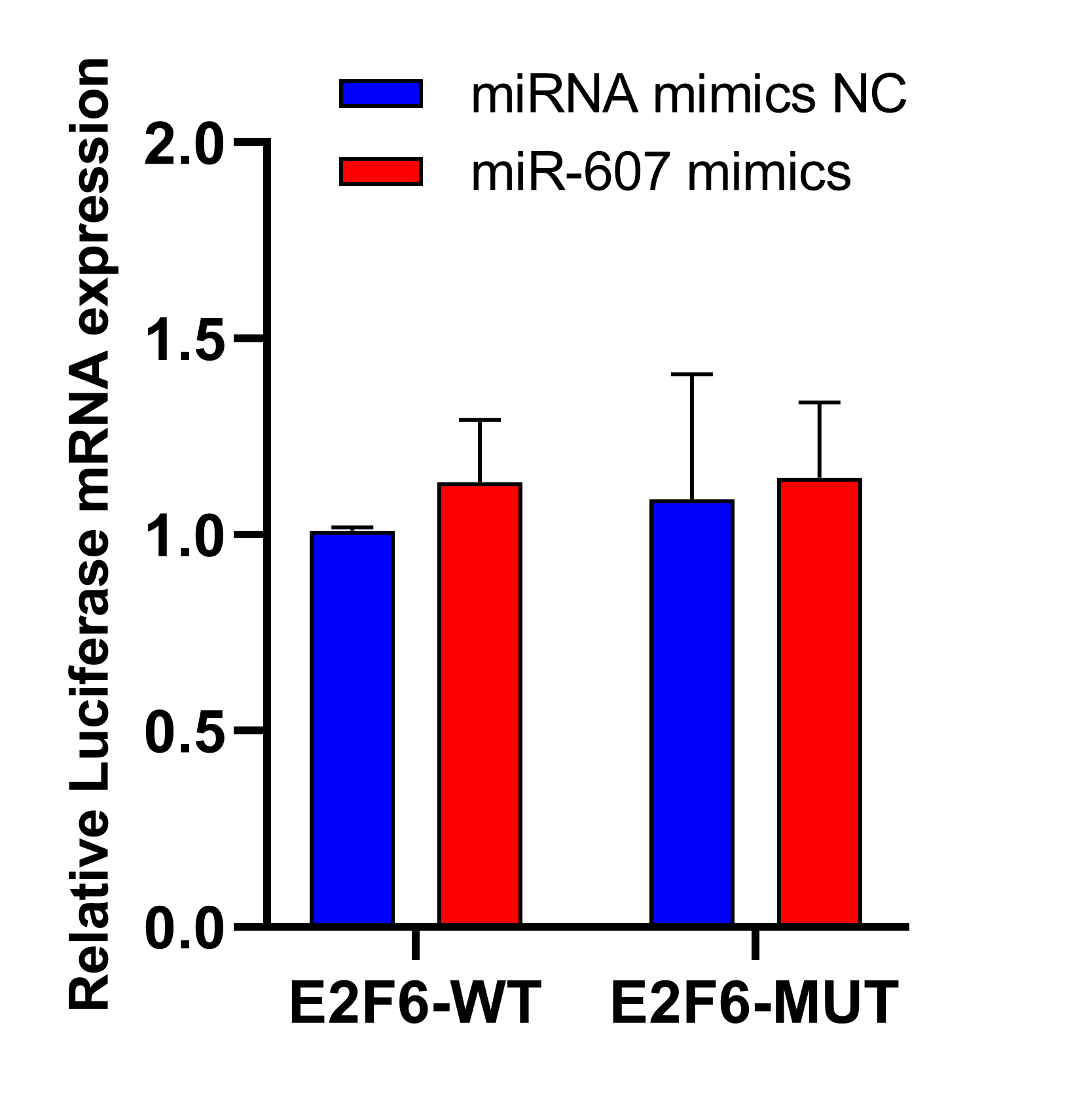

Supplement: Supplementary Figure 1 — The effect of miRNA mimics NC or miR-607 mimics on the expression of luciferase mRNA with wild-type or mutant binding sites. [file Image_1.tif]

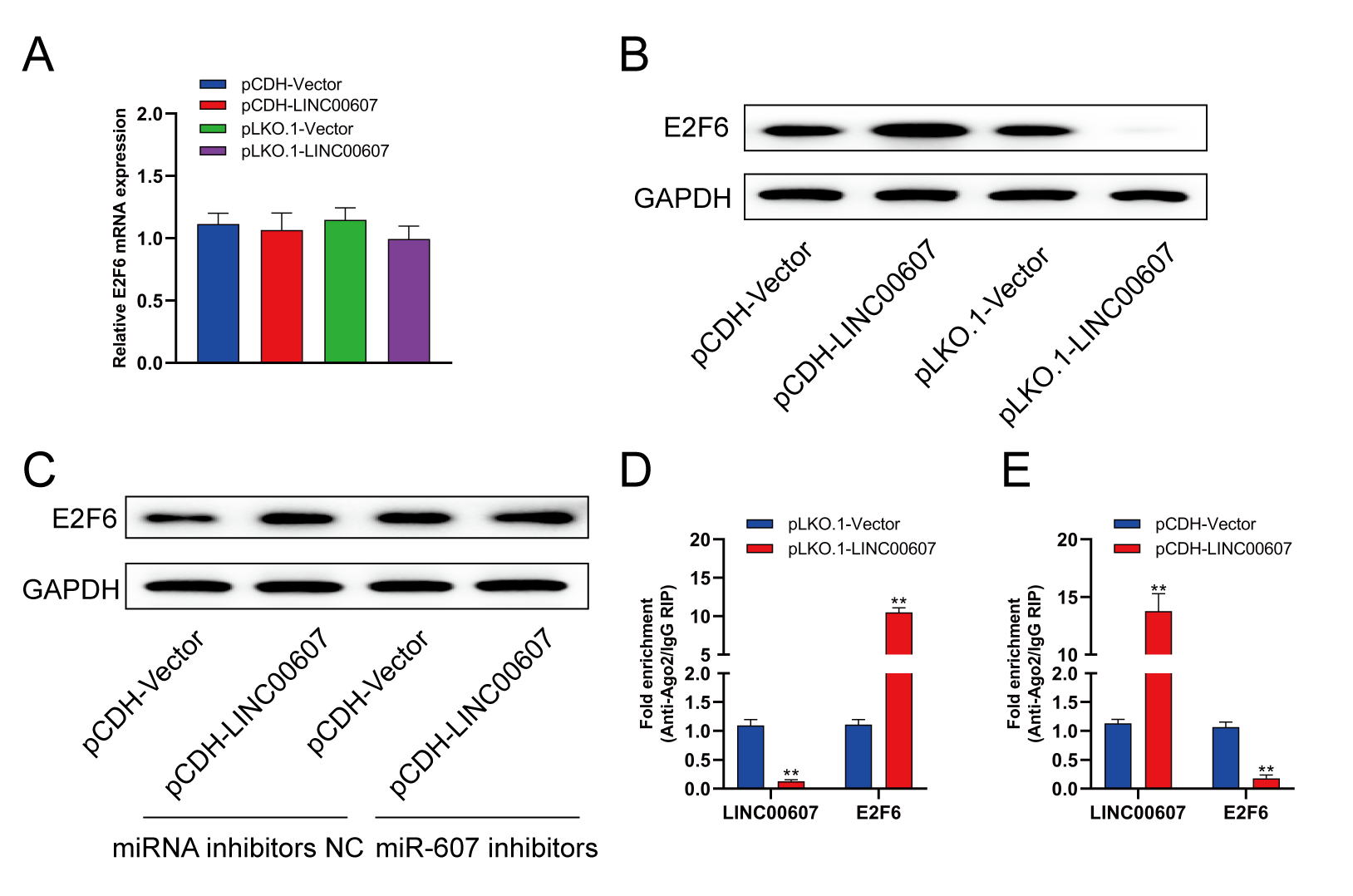

Supplement: Supplementary Figure 2 — LINC00607 regulated E2F6 expression via miR-607. (A) RT-PCR detected E2F6 mRNA expression in U2OS cells with LINC00607-overexpressing/knockdown plasmids. (B) Western blot detected the expression of E2F6 protein with LINC00607-overexpressing/knockdown in U2OS cells. (C) Western blot detected the expression of E2F6 protein in U2OS cells with LINC00607-overexpressing plasmids and miR-607 inhibitors. (D) RIP assay of the enrichment of Ago2 on LINC00607 and E2F6 transcripts relative to IgG in U2OS cells transfected with either pCDH-Vector or pCDH-LINC00607. (E) RIP assay of the enrichment of Ago2 on LINC00607 and E2F6 transcripts relative to IgG in U2OS cells transfected with either pLKO.1-Vector or pLKO.1-LINC00607. Statistical analysis was conducted using Student’s t-test. Values are expressed as mean ± SD compared with the control group. *p < 0.05, **p < 0.01. [file Image_2.tif]

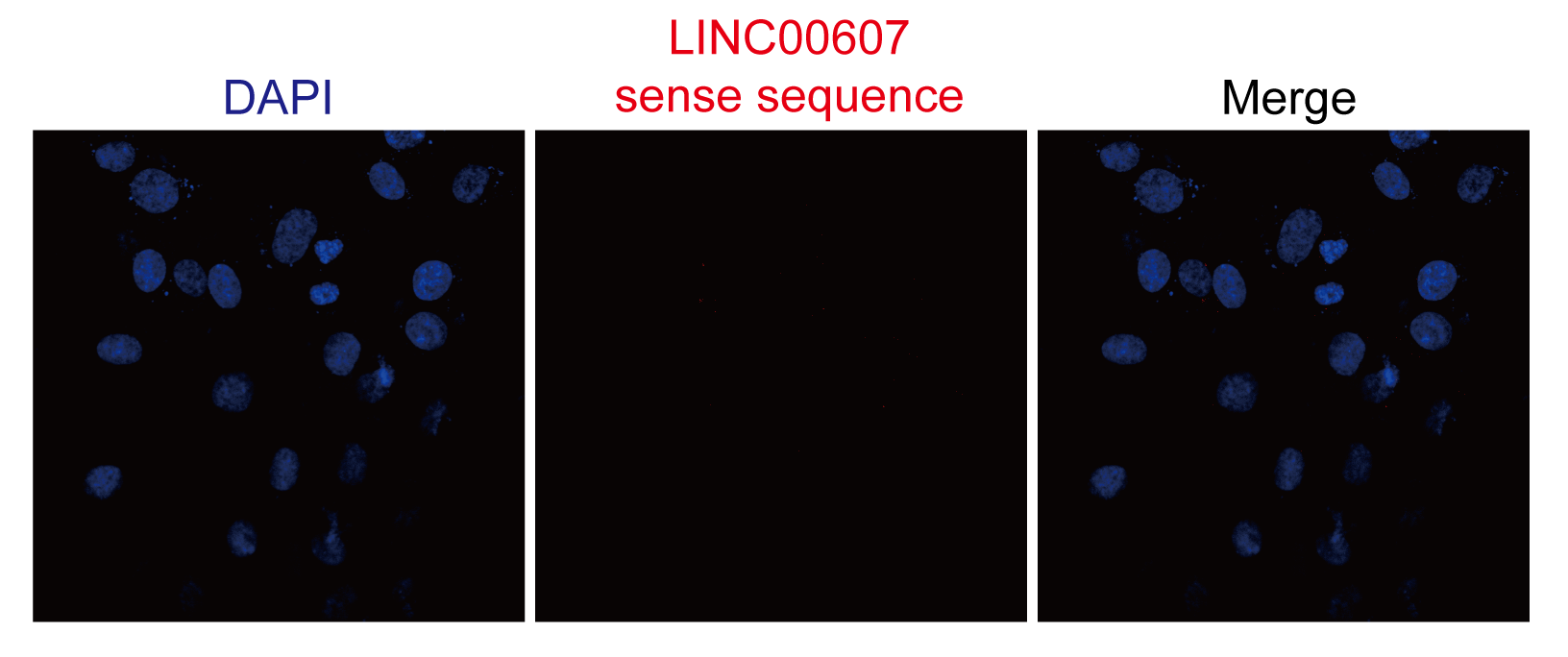

Supplement: Supplementary Figure 3 — Fluorescence in situ hybridization (FISH) for LINC00607 using sense sequence in U2OS. [file Image_3.tif]

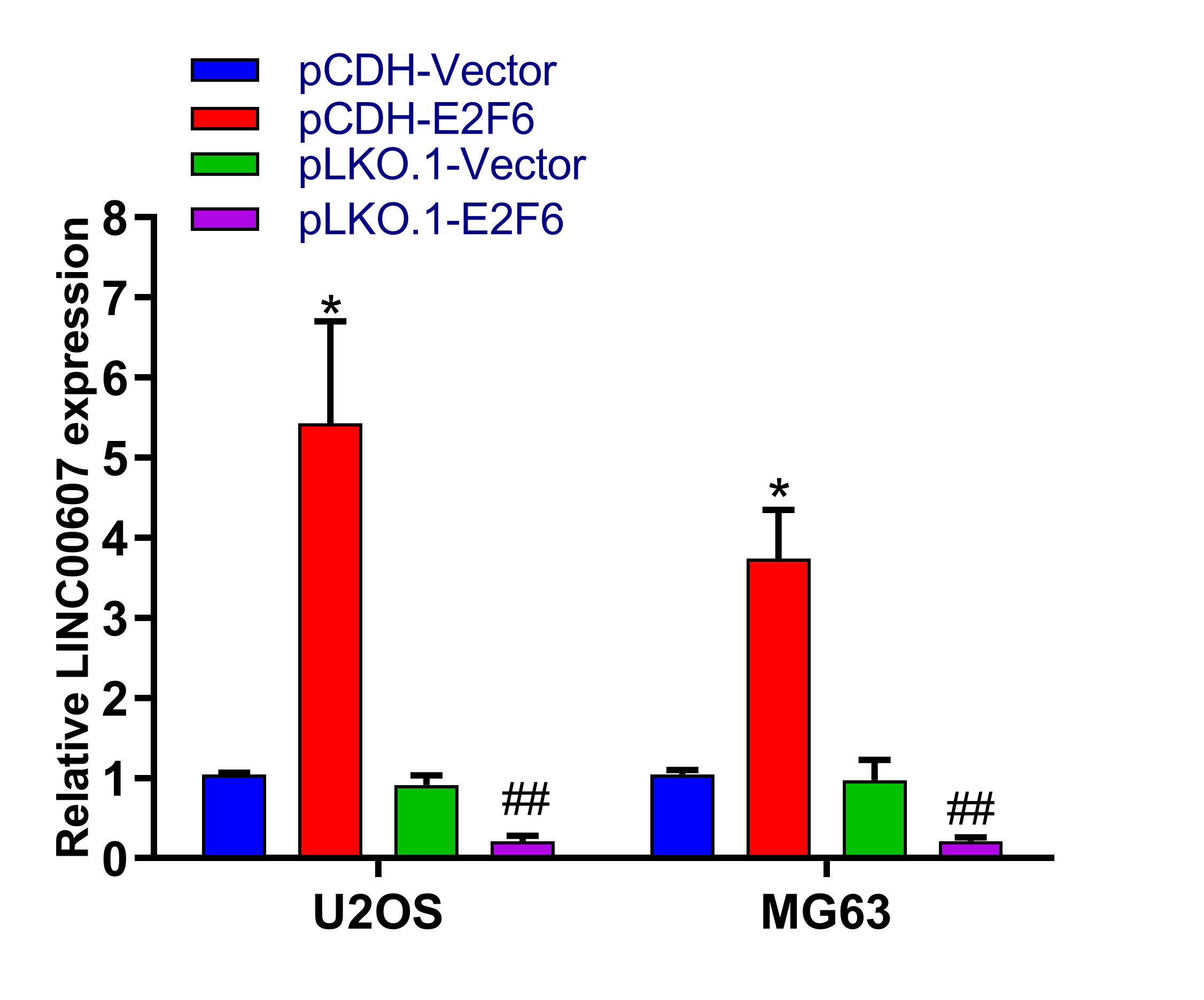

Supplement: Supplementary Figure 4 — E2F6 regulates the expression of LINC00607 in U2OS and MG63 cells. [file Image_4.tif]

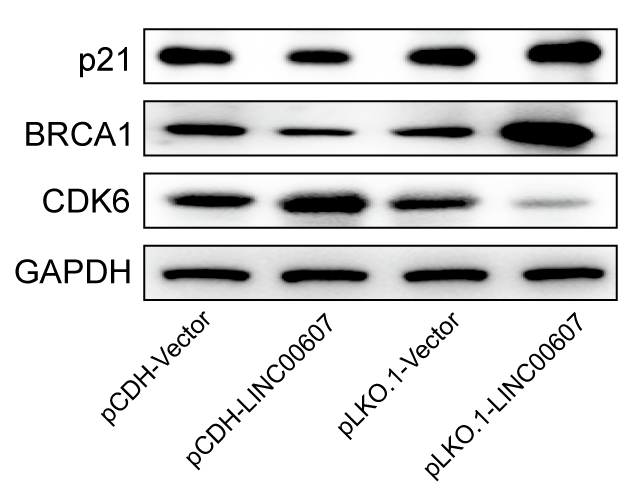

Supplement: Supplementary Figure 5 — LINC00607 regulates the expression of p21, BRCA1, and CDK6. [file Image_5.tif]
